# Association of the dietary inflammatory index with phenotypic age in the United States adults

**DOI:** 10.4178/epih.e2023051

**Published:** 2023-05-04

**Authors:** Mengzi Sun, Jiaxin Fang, Wenhui Gao, Yue He, Yanan Ma, Lina Jin

**Affiliations:** 1 Department of Epidemiology and Biostatistics, School of Public Health, Jilin University, Changchun, China; 2 Department of Biostatistics and Epidemiology, School of Public Health, China Medical University, Shenyang, China

**Keywords:** Elderly, National Health and Nutrition Examination Survey, Inflammation, Aging

## Abstract

**OBJECTIVES:**

One of the underlying mechanisms of aging is chronic inflammation, which has been closely associated with daily diet. Phenotypic age (PhenoAge) has been used as an index to track the aging process before diseases show clinical symptoms. The present study aimed to explore the association between the dietary inflammatory index (DII) and PhenoAge.

**METHODS:**

In total, 9,275 adults aged 20 years old and over in the National Health and Nutrition Examination Survey were involved in this study. Dietary patterns were classified as pro-inflammatory or anti-inflammatory according to the DII. PhenoAge was regarded as a continuous variable, and linear regression was used to explore its association with dietary inflammation. Stratified analyses by sex, age, race, physical exercise, smoking status, drinking status, and body mass index were used to test the sensitivity of these associations.

**RESULTS:**

The median value of PhenoAge was 38.60 years and 39.76 years for the participants with anti-inflammatory and pro-inflammatory diets, respectively. A pro-inflammatory diet was positively associated with PhenoAge (β=0.73; 95% confidence interval, 0.31 to 1.14), compared with participants who had an anti-inflammatory diet. There was an interaction between dietary inflammation and age for PhenoAge (p_interaction_<0.001). The strength of the association between a pro-inflammatory diet and PhenoAge was stronger as age increased.

**CONCLUSIONS:**

A pro-inflammatory diet was associated with a higher PhenoAge, and the association was strongest in the elderly. We recommended reducing dietary inflammation to delay phenotypic aging, especially for the elderly.

## INTRODUCTION

According to a 2019 report from the United Nations, 9% of people in the world are over 65 years of age, and by 2050 this number will rise to 16% [1]. The world will then experience an unsustainable burden of chronic diseases, which already extract a significant social and economic toll [2]. Aging involves changes in body composition, homeostatic mechanisms, energetics, and brain health over time. Therefore, aging could be reflected by a systematic analytical approach that integrates multiple biomarkers simultaneously, which could provide an opportunity to identify comprehensive biomarker signatures of aging [3,4]. Noteworthily, phenotypic age (PhenoAge) was developed as a novel multi-systembased aging measure capable of capturing mortality and morbidity risk in healthy individuals [5,6]. Previous studies have proven that PhenoAge could facilitate the identification of individuals at risk for various chronic diseases or causes of death [7].

Chronic inflammation is a significant risk factor for morbidity and mortality in the elderly and a common molecular pathway for most age-related diseases [8]. It has been reported that diet is closely associated with inflammation, and inflammation might be a bridge that links diet and chronic diseases [9,10]. The dietary inflammatory index (DII) is a literature-derived score developed to evaluate the inflammatory potential of the diet and link diet to inflammation [11]. Meanwhile, the DII could serve as a quantitative measure for assessing the relationships between diet and health outcomes [12]. A cohort study indicated that a higher DII (indicating more significant pro-inflammatory diet potential) was associated with an increased risk of incident dementia [13], and a cross-sectional study indicated that the DII was positively associated with cognitive impairment in the elderly [14]. The DII was also associated with frailty and 8-year mortality risk in adults of all ages by another cohort study [15]. Moreover, an association was also found between DII and leukocyte telomere length (LTL) [16]. A previous study pointed out that nutritional issues play a vital role in age-associated diseases and substantially contribute to morbidity, disability, and mortality [17]; thus, it is necessary to form better nutritional habits to improve health outcomes.

However, no studies have explored the association between DII and PhenoAge, especially for different age groups. In this study, we aimed to explore the association between dietary inflammation and aging via DII and PhenoAge in adults based on the National Health and Nutrition Examination Survey (NHANES), and we attempted to quantify the effect of a pro-inflammatory diet on aging. Furthermore, our results might provide a reference for specifying measurements for aging prevention.

## MATERIALS AND METHODS

### Study population and protocol approval

**Figure 1.**
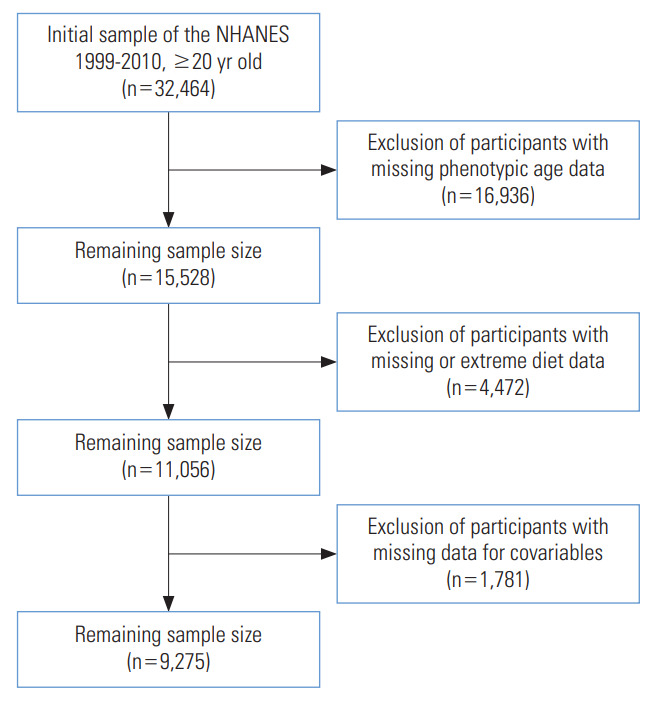
The flowchart of participants in this study. NHANES, National Health and Nutrition Examination Survey.

The NHANES is an ongoing cross-sectional survey that enrolls randomly selected participants for a comprehensive health screening every 2 years to generate a nationally representative sample [18]. A total of 32,464 participants aged over 20 were enrolled in NHANES from 1999 to 2010. After excluding participants who had missing data on PhenoAge, diet, and covariables, and who had extreme diet data, 9,275 participants were finally involved in the study. A flowchart is shown in Figure 1. We also described the characteristics of non-participants in Supplementary Material 1.

### Data measurement

#### Definition of PhenoAge

The PhenoAge calculation was proposed by Liu et al. [6] as a marker to track the aging process before diseases show clinical symptoms. PhenoAge was calculated using chronological age and 9 biomarkers (albumin, creatinine, glucose, log [C-reactive protein (CRP)], lymphocyte percent, mean cell volume, red blood cell distribution width, alkaline phosphatase, and white blood cell count). PhenoAge was selected through a Cox proportional hazards elastic net model for mortality based on 10-fold cross-validation [5,6], and PhenoAge was found to represent a person’s expected age within the population, consistent with a person’s estimated mortality hazard as a function of his/her profile of chemistry biomarkers [19]. The resulting final equation [20] to calculate PhenoAge is shown below:


$$PhenoAge=141.50+\frac{\ln\left[-0.00553\times ln(1-M)\right]}{0.09165}$$

Where


$$M=1-exp(\frac{-1.51714\times exp\;(xb)}{0.0076927})$$

xb=-19.907-0.0336×albumin+0.0095×creatinine+0.1953×glucose+0.0954×In(CRP)-0.0120×lymphocyte percent+0.0268×mean cell volume+0.3306×red cell distribution width+0.00188×alkaline phosphatase+0.0554×white blood cell count+0.0804×chronological Age

#### Definition of the DII

We used the revised version of the DII calculation developed by Shivappa et al. [11], and the specific algorithm has been detailed in a previous study. In this study, 26 nutrients were used to calculate the DII, including alcohol, vitamin B12/B6, β-carotene, caffeine, carbohydrate, cholesterol, total fat, fiber, folic acid, iron, magnesium, zinc, selenium, monounsaturated fatty acids, niacin, n-3 fatty acids, n-6 fatty acids, protein, polyunsaturated fatty acids, riboflavin, saturated fat, thiamin, and vitamins A/C/E. Importantly, even if fewer than 30 nutrients are applied, the DII can still be calculated. Participants were divided into those with an anti-inflammatory diet (DII<0) and those with a pro-inflammatory diet (DII≥0) [21].

#### Covariate assessment

Body mass index (BMI) groups were defined into 3 categories: underweight and healthy weight (BMI < 25.0 kg/m^2^), overweight (25.0≤ BMI< 30.0 kg/m^2^), and obese (BMI ≥ 30.0 kg/m^2^) [22]. The physical activity level was measured with the Global Physical Activity Questionnaire [23] and divided into 3 groups (“inactive,” “moderate,” and “vigorous”) based on self-reported questions. Smoking status was divided into 3 categories: Non-smokers were defined as those who never smoked or smoked fewer than 100 cigarettes in their lifetime; former smokers were defined as those who had smoked at least 100 cigarettes but did not smoke now; and current smokers were defined as participants who had at smoked least 100 cigarettes and reported a non-zero number of cigarettes per day in the past 30 days [24]. The NHANES defined 1 alcohol-based drink as 12 ounces of beer, 4 ounces of wine, or 1 ounce of liquor. Drinking status was divided into 3 categories: nondrinkers were defined as participants who had consumed fewer than 12 alcohol-based drinks in the past year or lifetime; former drinkers were defined as participants who had consumed at least 12 drinks in their lifetime but not in the past year; and current drinkers were defined as participants who had at least 12 drinks in the past year and reported a non-zero number of drinks per week [24]. Moreover, the educational level was categorized into 3 groups: under high school, high school, and college degree or above. Income was measured by the ratio of family income to the poverty threshold.

### Statistical analysis

The mean and standard error (SE) were used to describe continuous variables, and the unweighted frequency and weighted percentage were used to describe categorical variables. Multivariate linear regression was used to evaluate the associations between dietary inflammation and PhenoAge with adjustments. Sensitivity analyses were conducted via stratified analyses, and p-values for interaction between dietary inflammation and each stratified variable were also tested. All statistical analyses were conducted using SPSS version 24.0 (IBM Corp., Armonk, NY, USA) and R version 4.1.0 (R Foundation for Statistical Computing, Vienna, Austria), and the packages “forestplot” [25] and “survey” [26] were used. A 2-sided p-value < 0.05 was considered significant. All the analyses were performed using the complex sampling weight of NHANES.

### Ethics statement

The institutional review board approved the protocols of the NHANES of the National Center for Health Statistics, Centers for Disease Control and Prevention. Written informed consent was obtained from each participant before participation in this study. The responsible committee of the Ethics Committee of the National Center for Health Statistics Research Ethics Review Board has approved the research protocol (ID: #98-12, #2005-06).

## RESULTS

Table 1 shows the basic characteristics of the 9,275 participants, of whom 4,496 had an anti-inflammatory diet and 4,779 had a pro-inflammatory diet. The median PhenoAge was 39.04 years, 38.60 years, and 39.76 years for the total participants, the participants with an anti-inflammatory diet, and the participants with a pro-inflammatory diet, respectively.

We conducted univariate linear regression to test the relationships of variables with PhenoAge in adults, as shown in Table 2. The variables showed statistically significant associations with PhenoAge except for sex and income. Table 3 shows the association between dietary inflammation and PhenoAge in adults according to multivariate linear regression. Compared to participants with an anti-inflammatory diet, those with a pro-inflammatory diet had a PhenoAge in model 1 (pro-inflammatory diet: β= 1.78; 95% confidence interval [CI], 1.43 to 2.14). Similar results were observed in model 2 (pro-inflammatory diet: β= 1.38; 95% CI, 1.05 to 1.71) and model 3 (pro-inflammatory diet: β= 0.73; 95% CI, 0.31 to 1.14). A larger coefficient indicated a greater risk of the pro-inflammatory diet for higher PhenoAge.

**Figure 2.**
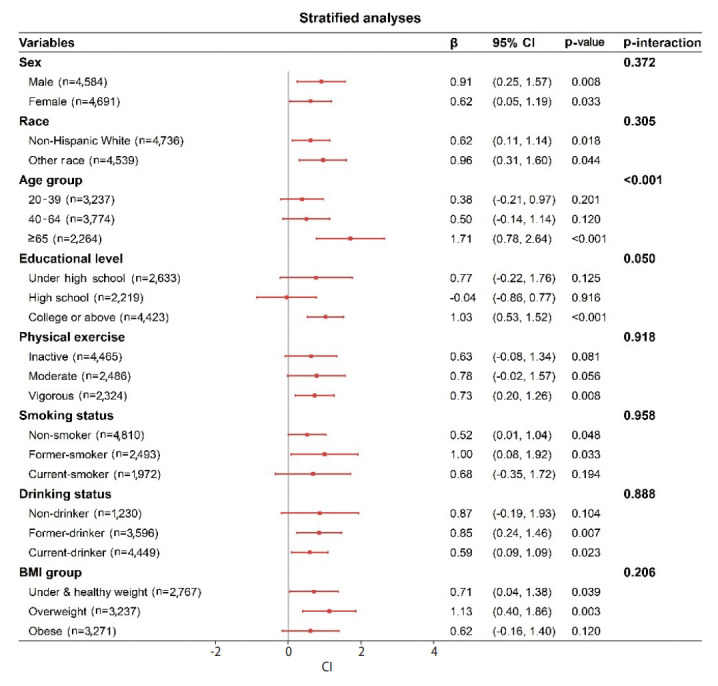
Forest plot of stratified analyses of the associations between a pro-inflammatory diet and phenotypic age in adults. Adjusted for age (years), sex (male, female), race (non-Hispanic White, other), BMI (kg/m^2^), energy intake (kcal), smoking status (non-smoker, current smoker, former smoker), drinking status (non-drinker, current drinker, former drinker), and physical exercise (inactive, moderate, vigorous), educational level (under high school, high school, college or above), and ratio of family income to the poverty threshold. CI, confidence interval; BMI, body mass index.

As shown in the forest plot in Figure 2, the positive association between a pro-inflammatory diet and PhenoAge was robust after a stratified analysis. Noteworthily, there was an interaction between dietary inflammation and age on PhenoAge (p_interaction_< 0.001). The association between a pro-inflammatory diet and PhenoAge was stronger as age increased. Moreover, we did not observe statistically significant interactions of dietary inflammation with sex, race, physical exercise, smoking status, drinking status, or BMI group on PhenoAge.

## DISCUSSION

In this study, we investigated the association between dietary inflammation and PhenoAge, and we found that a pro-inflammatory diet was significantly associated with a higher PhenoAge. Additionally, an interaction was found between a pro-inflammatory diet and age, with the strongest association between a pro-inflammatory diet and PhenoAge found in the elderly.

No previous cohort or cross-sectional study has examined the relationship between the DII and PhenoAge. Our study was the first to do so, to the best of our knowledge. PhenoAge has been proven to be more than a measure of disease or morbidity; instead, it may be a marker that tracks the effect of aging before diseases become clinically evident [6]. Meanwhile, PhenoAge could capture pre-clinical aging and future morbidity/mortality risk, facilitate the evaluation of intervention efficacy, and avoid the need for decades of follow-up [27]. Based on the positive association observed between a pro-inflammatory diet and PhenoAge, we recommend following an anti-inflammatory diet to lower PhenoAge in the elderly, thereby preventing the adverse consequences of aging.

Aging is a ubiquitous and complex phenomenon, and epidemiological evidence has indicated that elevated inflammatory biomarkers, including CRP and interleukin (IL)-6 could reflect a mild inflammatory state associated with many aging phenotypes [8]. “Inflamma-aging” is a common finding in aging and age-related disease that involves dysregulation of the cytokine network and its homeostasis [28]. The major pro-inflammatory cytokines, such as IL-6, tumor necrosis factor-alpha, and IL-1α, contribute significantly to inflammatory aging in healthy elderly individuals [29]. Moreover, based on the direct effect of pro-inflammatory cytokines on the muscle catabolic and anabolic signaling pathways, inflammation may contribute to the development of sarcopenia [30].

Diet is an important and potentially easily modifiable risk factor for chronic disease [31]. There has been extensive interest in how dietary strategies can improve immunity in the elderly, and the nutritional approach is particularly suitable for the aging population [32]. Several previous studies have explored the associations between diet and aging, including caloric restriction diets [33,34], different dietary patterns such as the plant-based Mediterranean diet [17,35], and specific nutrients such as long-chain omega-3 fatty acids (docosahexaenoic acid and eicosapentaenoic acid) [36]. The lower cardiovascular disease risk found in populations with high adherence to the Mediterranean diet may be partly explained by the consumption of the Mediterranean diet reducing the postprandial inflammatory response in mononuclear cells compared with saturated fatty acid-rich and carbohydrate/polyunsaturated fatty acid-rich diets in older adults [37]. Another study proved that plant-based dietary patterns were associated with lower oxidative stress and inflammation levels, which may provide a reasonable approach for chronic disease prevention [38]. Although previous studies have focused on dietary patterns and different age-related diseases, they all support the association between a healthy diet and longevity.

Moreover, dietary inflammation has been found to be positively associated with cognitive impairment, frailty, cardiometabolic risk, and other age-related diseases in previous studies [14,15,39]. Interestingly, some anti-aging nutrients are anti-inflammatory, such as vitamins A, D, E, and K and omega-3 fatty acids [40]. A previous study indicated that pro-inflammatory diets, which are typically high in refined grains, whole-fat dairy, red eat, total fat, and saturated fat, are positively associated with higher levels of inflammatory biomarkers [41]. It was reported that a higher DII was associated with a shorter LTL, an important aging biomarker [16]. The inflammatory potential of the diet was also related to incident frailty, slow walking, lower muscle mass, and poorer muscle function in older adults [42,43].

There are some strengths and weaknesses of current research. As for the advantages, firstly, it was the first study focused on the DII and PhenoAge, which are combined indicators of dietary inflammation and aging, respectively. Secondly, our study could provide evidence to support preventing aging from a dietary perspective. Thirdly, our study was based on the NHANES, a nationally representative survey. As for the weaknesses, firstly, this was a cross-sectional study and might not enable robust causal inferences. Secondly, the study population was from the United States, and the conclusions may not be generalized to other populations. Thirdly, the 24-hour recall diet data may have been subject to recall bias. Furthermore, we need to expand the cohort study’s sample size to explore in-depth associations between dietary inflammation and PhenoAge and how dietary inflammation affects aging in the general population. Finally, the age of participants was slightly lower than the age of non-participants, which may have caused selection bias; thus, the PhenoAge may have been underestimated. However, we performed a sensitivity analysis to reanalyze the imputed data, and consistent findings were obtained.

## DATA AVAILABILITY

The data that support the findings of this study are openly available at https://www.cdc.gov/nchs/nhanes/. Information from NHANES is made available through an extensive series of publications and articles in scientific and technical journals. For data users and researchers throughout the world, survey data are available on the Internet and on easy-to-use CD-ROMs.

## Figures and Tables

**Table 1.** Baseline characteristics of participants in elderly in NHANES 1999-2010 by inflammatory diet

Characteristics		Total (n=9,275)	Anti-inflammatory diet (n=4,496)	Pro-inflammatory diet (n=4,779)
Sex				
	Male	4,584 (49.4)	2,638 (59.4)	1,946 (38.0)
	Female	4,691 (50.6)	1,858 (40.6)	2,833 (62.0)
Race				
	Non-Hispanic White	4,736 (73.3)	2,546 (77.8)	2,190 (68.3)
	Other race	4,539 (26.7)	1,950 (22.2)	2,589 (31.7)
Age (yr)		45 (33, 59)	45 (34, 58)	45 (31, 60)
Age group (yr)				
	20-39	3,237 (38.9)	1,610 (37.7)	1,627 (40.2)
	40-64	3,774 (44.3)	1,896 (46.9)	1,878 (41.4)
	≥65	2,264 (16.8)	990 (15.4)	1,274 (18.4)
Educational level				
	Under high school	2,633 (18.3)	971 (13.3)	1,662 (24.0)
	High school	2,219 (25.0)	989 (21.7)	1,230 (28.7)
	College degree or above	4,423 (56.7)	2,536 (65.1)	1,887 (47.3)
Ratio of family income to poverty		3.11 (1.58, 5.00)	3.65 (2.04, 5.00)	2.40 (1.27, 4.20)
Physical exercise				
	Inactive	4,465 (42.0)	1,934 (38.3)	2,531 (46.3)
	Moderate	2,486 (28.6)	1,266 (28.9)	1,220 (28.2)
	Vigorous	2,324 (29.4)	1,296 (32.8)	1,028 (25.4)
Smoking status				
	Non-smoker	4,810 (50.7)	2,401 (52.8)	2,409 (48.4)
	Former smoker	2,493 (26.2)	1,311 (29.1)	1,182 (22.9)
	Current smoker	1,972 (23.1)	784 (18.1)	1,188 (28.8)
Drinking status				
	Non-drinker	1,230 (10.4)	454 (7.7)	776 (13.6)
	Former drinker	3,596 (41.3)	1,814 (43.0)	1,782 (39.3)
	Current drinker	4,449 (48.3)	2,228 (49.4)	2,221 (47.1)
BMI (kg/m^2^)		27.35 (23.91, 31.77)	26.94 (23.75, 31.04)	27.80 (24.05, 32.55)
BMI group				
	Underweight and healthy weight	2,767 (33.2)	1,423 (35.0)	1,344 (31.1)
	Overweight	3,237 (33.2)	161 8(34.3)	1,619 (32.1)
	Obese	3,271 (33.6)	1,455 (30.7)	1,816 (36.8)
Energy intake (kcal)		2,034.0 (1,551.5, 2,644.5)	2,449.0 (1,983.0, 3,075.0)	1,608.5 (1,289.2, 2,030.0)
PhenoAge (yr)		39.04 (25.92, 53.49)	38.60 (26.28, 51.65)	39.76 (25.67, 55.61)

Values are presented as median (percentile 25, percentile 75) or number (%). NHANES, National Health and Nutrition Examination Survey; BMI, body mass index; PhenoAge, phenotypic age.

**Table 2.** Univariate linear regression of variables on the phenotypic age in adults in NHANES 1999-2010

Variables	β (95% CI)	p-value
Dietary inflammation		
Anti-inflammation	Reference	
Pro-inflammation	1.37 (0.29, 2.46)	0.014
Sex		
Female	Reference	
Male	-0.82 (-1.83, 0.18)	0.107
Race		
Non-Hispanic White	Reference	
Other race	-5.37 (-4.14, -6.59)	<0.001
Age (yr)	1.06 (1.05, 1.07)	<0.001
Age group (yr)		
20-39	Reference	
40-64	22.29 (21.60, 22.99)	<0.001
≥65	46.55 (45.84, 47.26)	<0.001
Educational level		
Under high school	Reference	
High school	-1.62 (-3.68, 0.44)	0.121
College or above	-5.37 (-7.09, -3.66)	<0.001
Ratio of family income to poverty	0.06 (-0.34, 0.45)	0.765
Physical exercise		
Inactive	Reference	
Moderate	-1.83 (-3.04, -0.62)	0.004
Vigorous	-10.22 (-11.46, -8.98)	<0.001
Smoking status		
Non-smoker	Reference	
Former smoker	10.52 (9.20, 11.84)	<0.001
Current smoker	-0.65 (-2.06, 0.76)	0.363
Drinking status		
Non-drinker	Reference	
Former drinker	-3.83 (-6.04, -1.63)	0.001
Current drinker	-4.85 (-7.15, -2.55)	<0.001
BMI (kg/m^2^)	0.51 (0.43, 0.58)	<0.001
BMI group		
Underweight and healthy weight	Reference	
Overweight	6.38 (5.19, 7.57)	<0.001
Obese	8.30 (7.11, 9.50)	<0.001
Energy intake	-0.00 (-0.00, -0.00)	<0.001

NHANES, National Health and Nutrition Examination Survey; CI, confidence interval; BMI, body mass index.

**Table 3.** Multivariate linear regression of dietary inflammation on the phenotypic age in the elderly in NHANES 1999-2010 in different models^1^

Variables	No. of events	Model I	Model II	Model III
Dietary inflammation				
Anti-inflammation	4,496	Reference	Reference	Reference
Pro-inflammation	4,779	1.78 (1.43, 2.14)	1.38 (1.05, 1.71)	0.73 (0.31, 1.14)

Values are presented as β (95% confidence interval). NHANES, National Health and Nutrition Examination Survey; BMI, body mass index.

1 Model I: adjusted for age (years) and sex (male, female); Model II: adjusted for age (years), sex (male, female), race (non-Hispanic White, other), and BMI (kg/m^2^); Model III: adjusted for age (years), sex (male, female), race (non-Hispanic White, other), BMI, energy intake (kcal), smoking status (non-smoker, current smoker, former smoker), drinking status (non-drinker, current drinker, former drinker), physical exercise (inactive, moderate, vigorous), educational level (under high school, high school, college or above), and ratio of family income to the poverty threshold.

## SUPPLEMENTARY MATERIALS

Supplement Material 1. Comparing the characteristics between participants and non-participants n(%).
