# Supplementary material for: Association of the dietary inflammatory index with phenotypic age in the United States adults
Source: Epidemiol Health. 2023 May 4;45:e2023051. doi: 10.4178/epih.e2023051 (PMC10593589; doi:10.4178/epih.e2023051)
Supplement: Supplement Material 1. — Comparing the characteristics between participants and non-participants n(%). [file epih-45-e2023051-Supplementary-1.docx]

Supplementary Material 1. Comparing the characteristics between participants and non-participants n(%).

| Variables | Total | Participants | Non-Participants |
| --- | --- | --- | --- |
| Sex (N=32464) |  |  |  |
| Male | 15526(47.8) | 4584(49.4) | 15526(47.2) |
| Female | 16938(52.2) | 4691(50.6) | 16938(52.8) |
| Race (N=32464) |  |  |  |
| Non-Hispanic White | 15993(49.3) | 4736(51.1) | 11257(48.5) |
| Other Race | 16471(50.7) | 4539(48.9) | 11932(51.5) |
| Age group (N=32464) |  |  |  |
| 20~39 | 11278(34.7) | 3237(34.9) | 8041(34.7) |
| 40~64 | 12569(38.7) | 3774(40.7) | 8795(37.9) |
| 65~ | 8617(26.6) | 2264(24.4) | 6353(27.4) |
| Educational level (N=32464) |  |  |  |
| Less than High School | 10081(31.1) | 2633(28.4) | 7448(32.2) |
| College or AA degree | 7700(23.8) | 2219(23.9) | 5481(23.7) |
| College or above | 14593(45.1) | 4423(47.7) | 10170(44.1) |
| BMI group (N=29991) |  |  |  |
| Under & health weight | 9133(30.5) | 2767(29.8) | 6366(30.7) |
| Overweight | 10486(35.0) | 3237(34.9) | 7249(35.0) |
| Obese | 10732(34.5) | 3271(35.3) | 7107(34.3) |
| Drinking status (N=27986) |  |  |  |
| Non-drinker | 4064(14.5) | 1230(13.3) | 2834(15.1) |
| Former-drinker | 9672(34.6) | 3598(48.0) | 6076(32.5) |
| Current-drinker | 14250(50.9) | 4449(38.7) | 9801(52.4) |
| Smoking status (N=32414) |  |  |  |
| Non-smoker | 17009(652.5) | 4810(51.9) | 12199(52.7) |
| Former-smoker | 8350(25.8) | 2493(26.9) | 5857(25.3) |
| Current-smoker | 7055(21.7) | 1972(21.2) | 5083(22.0) |
